# Affinity Maturation for Antibody Engineering: The Critical Role of Residues on CDR Loops of Antibodies in Antigen Binding

**DOI:** 10.3390/molecules30030532

**Published:** 2025-01-24

**Authors:** Mutsumi Yoshida, Masayuki Oda

**Affiliations:** Graduate School of Life and Environmental Sciences, Kyoto Prefectural University, 1-5 Hangi-cho, Shimogamo, Sakyo-ku, Kyoto 606-8522, Japan

**Keywords:** affinity maturation, antigen binding, crystal structure, thermal stability

## Abstract

During the course of affinity maturation, antibodies exhibit enhanced antigen-binding affinities by altering the amino acids in their variable regions. Understanding the structural basis of these antibodies can be beneficial for antibody engineering. We determined the crystal structures of single-chain Fv (scFv) antibodies against (4-hydroxy-3-nitrophenyl)acetyl, C6 and E11, which had undergone affinity maturation. Compared with germline-type antibodies, the affinity-matured antibodies with somatic hypermutation from Lys58 to Arg58 of the heavy chain located in the complementarity-determining region 2 (CDR2) seemed to be critical for increasing the antigen-binding affinity. E11 possessed a disulfide bond at the base of CDR3 in the heavy chain, which contributed to a further increase in its antigen-binding affinity compared with that of C6. In this study, we generated several mutant scFvs of C6 and E11 and analyzed their antigen-binding thermodynamics using isothermal titration calorimetry. The results indicated that the CDR conformations could adjust antigen-binding not only at the mutated sites but also at the surrounding residues. The analysis of folding thermodynamics showed that the stability of the affinity-matured antibodies was lower than that of the germline-type antibodies and remarkably increased upon strong antigen binding. The results also indicated that the structural dynamics of the affinity-matured antibodies were greater than those of the germline-type antibodies and decreased upon antigen binding.

## 1. Introduction

**Figure 1:**
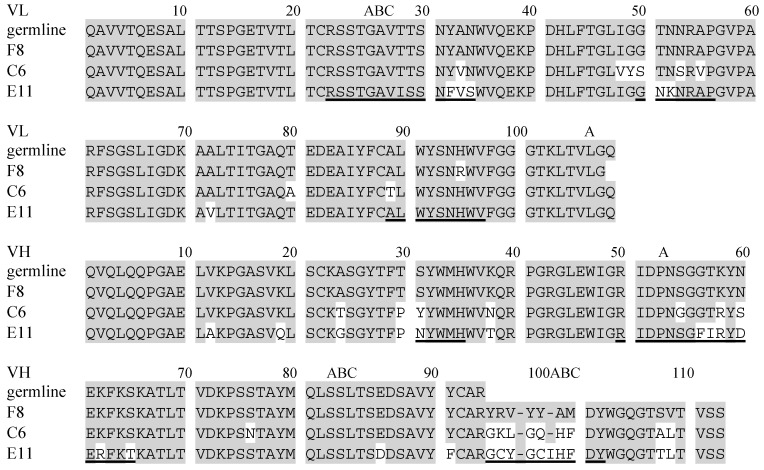
The sequence alignments of variable regions of anti-NP antibodies F8, C6, and E11. The gray boxes indicate the sequence similarity to a germline-type antibody and F8. The CDR regions are underlined. The residue number and CDR regions are based on Kabat et al. (1991) [26].

Affinity maturation of antibodies is a process that enhances their antigen-binding affinity and the specificity of an antibody by somatic hypermutation (SHM) and VDJ recombination [1,2,3,4,5,6]. The antigen-binding properties of antibodies undergoing affinity maturation can provide information about their structure–activity relationship, which can be applied to antibody engineering [7,8,9]. To analyze the structural basis of affinity maturation, various haptens, such as (4-hydroxy-3-nitrophenyl)acetyl (NP), phosphorylcholine, and 2-phenyl oxazolone, have been used [10,11,12,13,14]. The equilibrium association constant (*K*_a_) of germline-type antibodies for haptens is as low as 10^5^ M^−1^, while that of affinity-matured antibodies is as high as 10^9^ M^−1^ [15,16,17,18]. Anti-NP antibodies obtained from C57BL/6 mice encoded by the gene segments VH186.2, DFL16.1, and JH2 showed an approximately ten-fold increase in affinity via SHM from Trp to Leu at position 33 of the heavy chain (W33L^H^) [19,20,21,22]. Keeping Trp33^H^, a Y95G^H^ mutation increased the antigen-binding affinity approximately 10^3^-fold [16,23]. The NP-binding affinity, *K*_a_, of F8 is 2.7 × 10^5^ M^−1^, while those of C6 and E11 are 3.3 × 10^7^ M^−1^ and 5.8 × 10^8^ M^−1^, respectively (Figure 1). The residue at 95^H^ was located at the V–D junction, where the terminal deoxynucleotidyl transferase added non-templated nucleotides [24,25]. The Y95G^H^ mutation increased the antigen-binding affinity, primarily by altering the conformation of the heavy-chain complementarity-determining region 3 of (H-CDR3).

We determined the high-resolution crystal structures of the single-chain Fv (scFv) antibodies C6 and E11 complexed with NP [27,28]. Both C6 and E11 are affinity-matured antibodies possessing Gly95^H^, and they contain 17 and 24 SHMs, respectively. Together with the crystal structure information of N1G9 [29], a germline-type anti-NP antibody, the residues at 50^H^, 58^H^, and 96^L^, directly formed hydrogen bonds with NP. As shown in Figure 1, the residues at 50^H^ and 96^L^ are well-conserved as Arg and Trp, respectively. The residues at 58^H^ are Lys in germline-type antibodies including N1G9 and Arg in affinity-matured antibodies such as C6 and E11. The residue at 58^H^ is located on H-CDR2, and the length of the hydrogen bond of Lys58^H^ in N1G9 is shorter than that of Arg58^H^ in C6 [27,29]. During the maturation from C6 to E11, a disulfide bond is introduced into H-CDR3 between Cys96^H^ and Cys100^H^, in addition to the insertion of Ile at 100a^H^ (Figure 1). Our recent analysis indicated that the disulfide bond contributed to the stabilization of H-CDR3, resulting in increased antigen-binding affinity [28].

In this study, we generated scFv mutants of C6, namely C6_R58K^H^, C6_Q100E^H^, C6_100aI^H^, and C6_Q100E^H^/100aI^H^, to analyze the roles of the respective residues on CDR loops in antigen binding. The residue at 58^H^ is located on H-CDR2 and is changed from Lys to Arg via SHM (Figure 1). The residue at 96^H^ is located on H-CDR3, and those of C6 and E11 are Lys and Cys, respectively (Figure 1). The residue Lys96^H^ of C6 has the potential to form a salt bridge with Glu100^H^ in C6_Q100E^H^, thereby stabilizing the conformation of H-CDR3, similar to the case of a disulfide bond between Cys96^H^ and Cys100^H^ of E11 [28]. As observed in E11, an Ile100a^H^ insertion in C6 might be necessary to form the salt bridge and stabilize the conformation of H-CDR3, leading to the generation of C6_Q100E^H^/100aI^H^, with C6_100aI^H^. To analyze the effects of the Ile insertion, an Ile100a^H^-deleted mutant of E11 scFv, E11_Δ100aI^H^, was also generated. We analyzed the binding thermodynamics of these scFv mutants to NP and (4-hydroxy-3,5-dinitrophenyl)acetyl (NNP) using isothermal titration calorimetry (ITC) [30,31]. Anti-NP antibodies have a unique antigen-binding specificity, referred to as heterocliticity [32,33], and their binding affinities for NP analogs, such as (4-hydroxy-3-iodo-5-nitrophenyl) acetyl (NIP) and NNP, are higher than those for NP [34]. We also analyzed the thermal stability of the scFvs generated in the absence or presence of NP and NNP antigens, using circular dichroism (CD) and differential scanning calorimetry (DSC) [35,36]. As protein stability is closely correlated with structural dynamics in solution, it can provide insight into the structural differences between the antigen-bound and antigen-unbound states of antibodies.

## 2. Results

**Figure 2:**
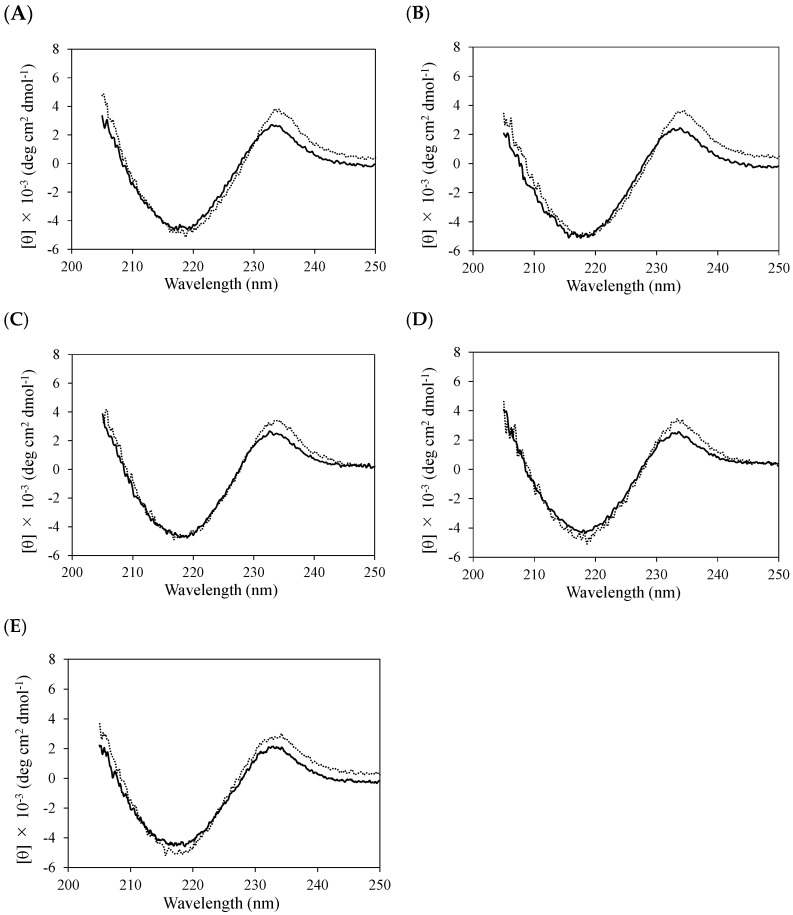
Far-UV CD spectra of scFvs of C6 and E11 mutants C6_R58K^H^ (**A**), C6_Q100E^H^ (**B**), C6_100aI^H^ (**C**), C6_Q100E^H^/100aI^H^ (**D**), and E11_Δ100aI^H^ (**E**) in the absence (black line) or presence (dotted line) of NP-Gly. The molar ratio of NP-Gly to scFv is 10:1 for C6_R58K^H^ (**A**) and C6_Q100E^H^ (**B**), 20:1 for C6_100aI^H^ (**C**) and E11_Δ100aI^H^ (**E**), and 30:1 for C6_Q100E^H^/100aI^H^ (**D**).

The anti-NP scFv antibodies overexpressed in *Escherichia coli* as inclusion bodies were solubilized with guanidine hydrochloride and were refolded by dilution in denaturing reagents. The refolded scFvs were purified using NP-conjugated bovine serum albumin (NP-BSA) and size-exclusion chromatography (SEC). The monomeric fractions of the scFvs were separated from the multimeric fractions using SEC. The far-UV CD spectra of the purified monomers showed a local minimum at 218 nm and a local maximum at 232 nm, indicating that the anti-NP scFv antibody was correctly folded (Figure 2 and Figure S1). The secondary structures remained almost unchanged upon antigen binding (Figure 2 and Figure S1).

**Figure 3:**
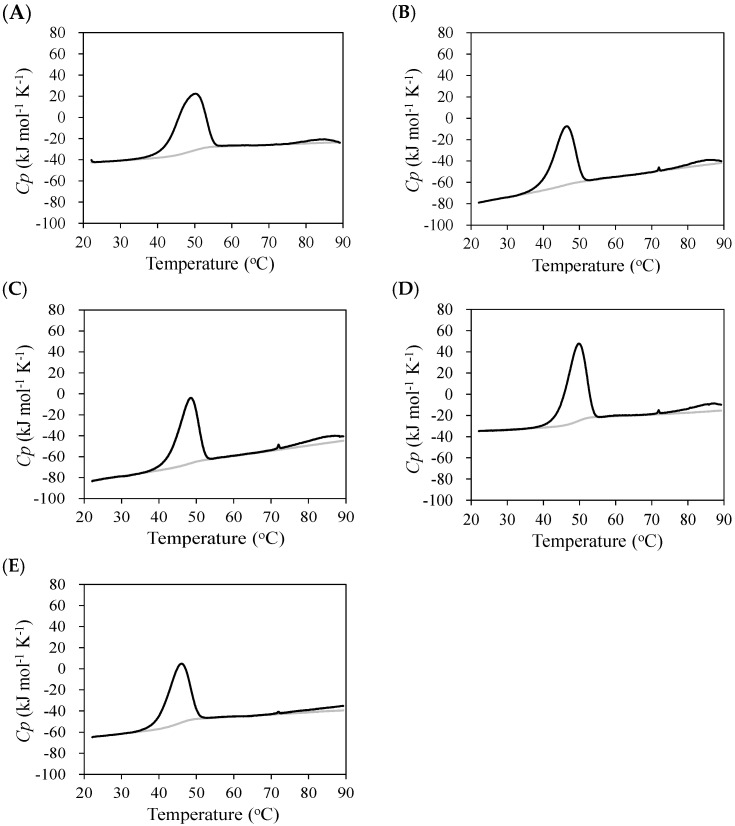
Heat capacity curves of scFvs of C6 and E11 mutants C6_R58K^H^ (**A**), C6_Q100E^H^ (**B**), C6_100aI^H^ (**C**), C6_Q100E^H^/100aI^H^ (**D**), and E11_Δ100aI^H^ (**E**) (black line). Heat capacity curves of buffer only used for background subtraction are also indicated (gray line).

The thermal stability of the anti-NP scFv antibodies was analyzed using CD and DSC (Figure 3). As the thermal unfolding was irreversible, a two-state model was applied to fit the data to provide the thermodynamic parameters (Table 1). Due to the irreversible unfolding, the denaturation temperatures, *T*_m_ and *T*_d_, were dependent on the scFv concentration. The present results showed that the *T*_m_ values for the scFv (0.04 mg mL^−1^) determined via CD were higher than the *T*_d_ values for scFv (1.0 mg mL^−1^) determined via DSC (Table 1). The stability of the C6 scFv decreased with the Q100E^H^ mutation but was restored with the insertion of Ile100a^H^. The insertion of Ile100a^H^ resulted in the decreased stability of C6 scFv. The deletion of Ile100a^H^ in the E11 scFv also resulted in decreased stability.

**Figure 4:**
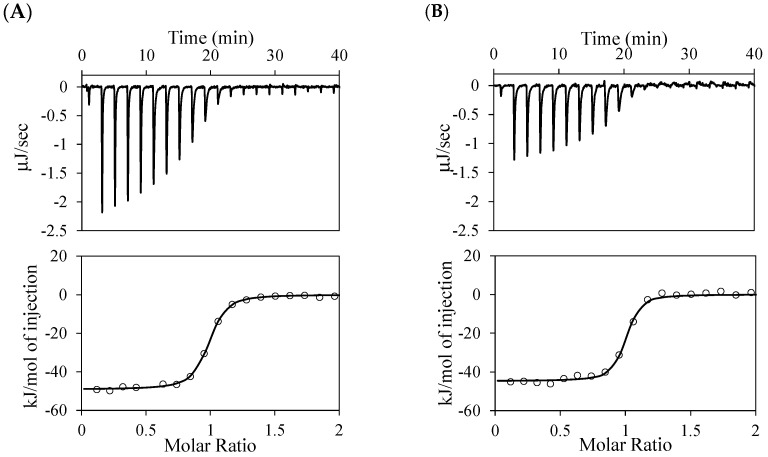

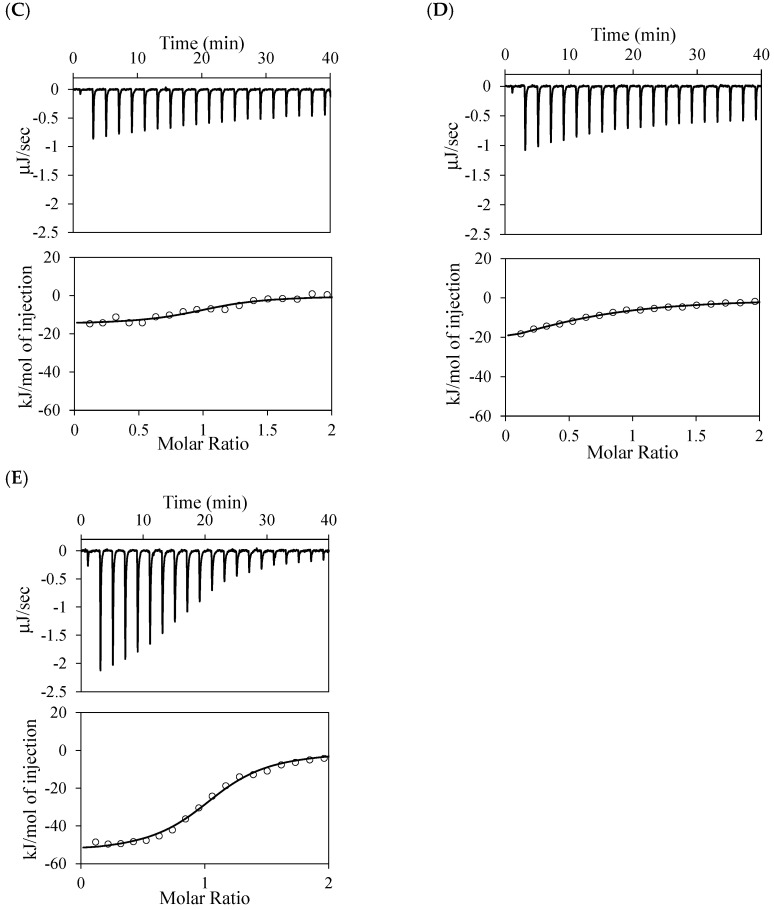
ITC profiles and diagram representing interactions between NP-Gly and scFvs: C6_R58K^H^ (**A**), C6_Q100E^H^ (**B**), C6_100aI^H^ (**C**), C6_Q100E^H^/100aI^H^ (**D**), and E11_Δ100aI^H^ (**E**). Antigen solution was titrated into the scFv solution (**upper**). The data points were obtained by integration of the peaks in titration profiles (**lower**), corrected for the dilution heat, and plotted against the molar ratio. The data were fitted using the nonlinear least-squares method.

The antigen-binding thermodynamics of the anti-NP scFv antibodies were analyzed using ITC (Figure 4 and Figure S2) and are summarized in Table 2. Heterocliticity was observed in all the scFvs analyzed. With the mutations of R58K^H^ and Q100E^H^, the binding affinities remained nearly unchanged with similar ∆*H* values. Upon the insertion of Ile100a^H^ into C6, the binding affinity considerably decreased, along with an increase in ∆*H*. The deletion of Ile100a^H^ in E11 resulted in decreased antigen-binding affinity, with NP binding of approximately 1/300 and NNP binding of approximately 1/20.

**Figure 5:**
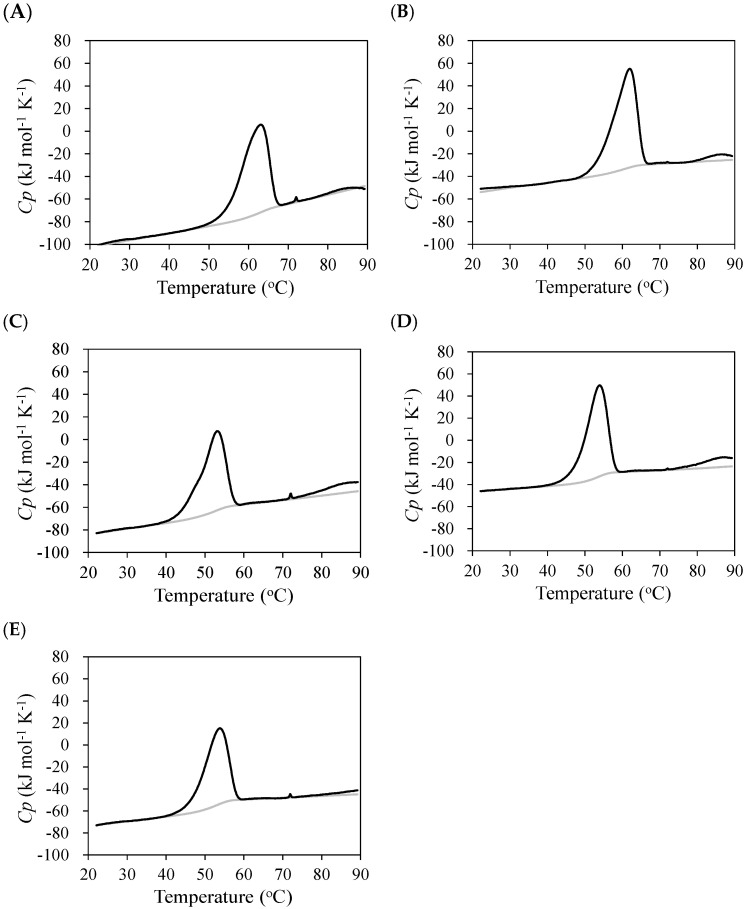
Heat capacity curves of scFvs of C6 and E11 mutants C6_R58K^H^ (**A**), C6_Q100E^H^ (**B**), C6_100aI^H^ (**C**), C6_Q100E^H^/100aI^H^ (**D**), and E11_Δ100aI^H^ (**E**) in the presence of NP-Gly in the molar ratio of 5:1 to scFv (black line). Heat capacity curves of buffer only used for background subtraction are also indicated (gray line).

The thermal stability of the anti-NP scFv antibodies in the presence of antigens was analyzed using CD and DSC (Figure 5 and Figure S3). The thermodynamic parameters are summarized in Table 3. The stability increased upon antigen binding. In comparing the NP and NNP binding, the increase in stability upon the NNP binding was found to be higher than that upon NP binding.

## 3. Discussion

Affinity maturation of antibodies is considered to be a type of evolution that enhances their antigen-binding affinity and can be utilized for antibody engineering. The affinity maturation of anti-NP antibodies was studied, and the structural basis of the affinity-matured antibodies, C6 and E11, was determined using high resolution crystal structures [27,28]. The antigen-binding affinity of C6 increased approximately 100-fold relative to that of the germline-type antibodies. Structural information indicated that the SHM from Lys58^H^ to Arg58^H^ seemed to be the most critical factor for enhancing affinity. However, the mutation of R58K^H^ in C6 had little effect on the affinities of both NP and NNP. One possible explanation for these results is that the conformation of H-CDR2 may be affected by other sites, in addition to the residue at 58^H^, and may alter the antigen-binding affinity. As observed in the crystal structure of NP-bound C6 [27], Arg58^H^ would have the advantage of forming multiple hydrogen bonds with both the hydroxyl and nitro groups of NP. The mutation of R58K^H^ may compensate for the reduced number of hydrogen bonds by decreasing the distance to the antigen, as observed in the crystal structure of N1G9 [29].

**Figure 6:**
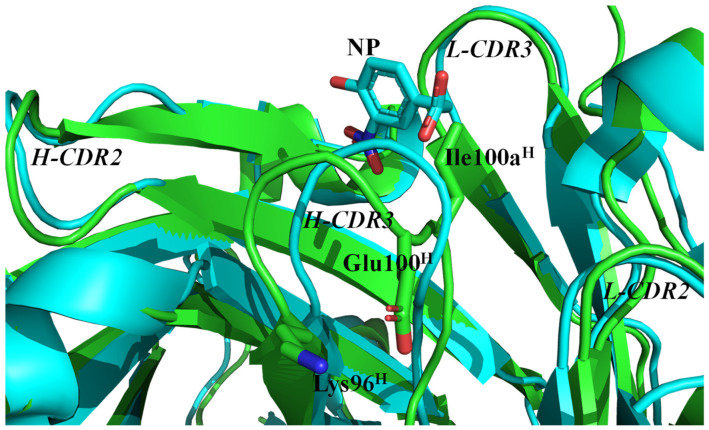
Structure model of C6_Q100E^H^/100aI^H^. The structure was modeled using AlphaFold3 (green) [37,38] and was superimposed on that of C6 in complex with NP (cyan) (PDB code; 6K4Z) [27]. The side chains of Lys96^H^, Glu100^H^, and Ile100a^H^ of C6_Q100E^H^/100aI^H^ were also indicated as stick models.

Mutations in H-CDR3 can generally affect antigen binding. Both the amino acid type and the CDR length can be altered via SHM and VDJ recombination. Upon the maturation of C6 to E11, the NP-binding affinity increased from approximately 10^7^ M^−1^ to 10^8^ M^−1^ [16]. A comparison of these antibodies can provide insights into the enhancement of affinity during the final stage of affinity maturation in the immune response. One of the most notable mutations in E11 is the introduction of a disulfide bond in H-CDR3. This was also observed in another affinity-matured antibody, E3. Both E11 and E3 showed the highest NP-binding affinities among the monoclonal antibodies obtained to date. In addition to Cys96^H^ and Cys100^H^, which are involved in disulfide bond formation, an Ile residue was inserted at site 100a^H^ (Figure 1). The antigen-binding affinity was reduced in the mutant scFv, E11_Δ100aI^H^, generated by removing Ile100a^H^ from E11 (Table 2). A preliminary crystal structure analysis of E11_Δ100aI^H^ revealed the formation of a disulfide bond between Cys96^H^ and Cys100^H^, indicating that the conformation of H-CDR3 changed upon the removal of Ile100a^H^. In the case of C6_Q100E^H^/100aI^H^, we expected that a salt bridge would form between Lys96^H^ and Glu100^H^, similar to the disulfide bond of E11; however, the antigen-binding affinity was found to be significantly decreased. The insertion of 100aI^H^ in C6 resulted in a decreased antigen-binding affinity with an increased ∆*H* value. In C6, the hydrogen bond network among Arg94^H^, Gln100^H^, and Asp101^H^ would stabilize the H-CDR3 conformation and would be weakened upon the insertion of 100aI^H^. The binding enthalpy change was reduced again in the double mutation of Q100E^H^ and 100aI^H^ (Table 2), indicating that the salt bridge was formed in C6_Q100E^H^/100aI^H^ but not in C6_100aI^H^. The salt bridge formation was supported by the results showing that the stability of C6_Q100E^H^/100aI^H^ was comparable to that of C6 and higher than those of C6_Q100E^H^ and C6_100aI^H^ (Table 1). The mutation of Q100E^H^ would disrupt the hydrogen bond network with Arg94^H^ and Asp101^H^. The double mutations of Q100E^H^/100aI^H^ would make it possible to form the salt bridge between Lys96^H^ and Glu100^H^ (Figure 6). However, the H-CDR3 conformation of C6_Q100E^H^/100aI^H^ would not fit well to the antigen, as in the case of E11. As reported previously [28], the mutation of C96K^H^/C100E^H^ in E11 altered the disulfide bond to the salt bridge, resulting in a slight decrease in the antigen-binding affinity, which was still at a high level of 6.04 × 10^7^ M^−1^.

The binding affinities of NNP to the scFvs analyzed in this study were higher than those of NP (Table 2). The additional nitro group involved in scFv binding enhances its affinity. As reported previously [34], the preference for NNP was higher in scFvs with low antigen-binding affinities than those with high affinities. The thermal stability of the scFvs complexed with NNP was higher than that of the scFvs complexed with NP. In general, the increased thermal stability of proteins upon ligand binding results not only from the ligand-binding energy but also from the increased folding energy of the protein. The NNP-bound scFv structure was more stable than the NP-bound scFv structure owing to the conformational change into a more rigid form. In the scFvs whose NNP-binding affinities exceeded 10^8^ M^−1^, the respective *T*_d_ values increased by >20 °C (Table 1 and Table 3). As described previously [39,40], the stability of the scFvs in the antigen-free form decreased with increasing affinity, reflecting a trade-off. For the structure–activity relationships, structural flexibility would be critical to achieve a high binding activity. SHM is a natural phenomenon of antibodies that enhances their antigen-binding affinity. The stability of affinity-matured antibodies in the antigen-free form was reduced and increased upon antigen binding. Compared with the antigen-bound forms, the stability of the scFv bound to NNP was higher than that bound to NP (Table 3). We can determine the structure–activity relationship from antibody evolution using scFv antibodies undergoing affinity maturation. As observed in N1G9 and E11, the 3D structure of the antibodies was similar in both the antigen-bound and antigen-unbound states [28,29], possibly due to the crystal structure showing the static and most stabilized form. Thermodynamic parameters include protein fluctuations in solution; the present results strongly indicate a difference in the structural dynamics between the antigen-bound and antigen-unbound states. An analysis of structural dynamics at high resolution will provide more quantitative information on the changes in binding affinity and stability, which may be useful in the fields of protein and antibody engineering [41,42].

## 4. Materials and Methods

### 4.1. Expression and Purification of scFv

The plasmid encoding scFv, a variable region of the light chain was connected to that of the heavy chain via a (G_4_S)_3_ linker, was transformed into *Escherichia coli* BL21 (DE3) codon plus, and the transformed cells were cultured in LB medium containing ampicillin (0.1 mg mL^−1^) at 37 °C. As described previously [28,41], scFvs were expressed in the insoluble fraction, solubilized using 6 M guanidine hydrochloride, and refolded by stepwise dilution in a solution containing urea from 4 M to 0 M, via 2 and 1 M. After removing the thioredoxin tag at the N terminus using thrombin (Mochida Pharmaceutical Co., Ltd., Tokyo, Japan), the isolated scFv was purified using an antigen column, in which NP-BSA was immobilized on the resin (NHS-activated sepharose 4; Cytiva, Tokyo, Japan). The scFv was further purified using SEC (HiPrep 26/600 Superdex 75 prep grade HR; Cytiva, Tokyo, Japan). The buffer containing purified scFvs was exchanged into phosphate-buffered saline (pH 7.4) using Ultra-4 (Merck, Darmstadt, Germany). The concentrations of scFvs were spectrophotometrically determined using molar absorption coefficients of 5.64 × 10^4^ M^−1^ cm^−1^ and 5.19 × 10^4^ M^−1^ cm^−1^ at 280 nm for C6 and E11 series, respectively.

### 4.2. CD Experiments

Far-UV CD spectra of scFvs (0.04 mg mL^−1^) were measured at 20 °C on a Jasco J-1100 spectropolarimeter (JASCO, Tokyo, Japan), as described previously [28]. The melting curves were recorded using CD values at 218 nm for each scFv in the absence or presence of antigens NP-Gly and NNP-Cap, with a heating rate of 1.0 °C min^−1^. The analysis of the transition curve to determine the thermal denaturation temperature (*T*_m_) was performed on the basis of a two-state transition model, as described previously [43].

### 4.3. ITC Experiments

ITC measurements were carried out using an iTC200 calorimeter (Malvern Panalytical, Tokyo, Japan), as described previously [28]. The solution of NP-Gly or NNP-Cap (100 or 200 μM) was titrated into the scFv solution (10 or 20 μM) at 25 °C. The heat for each injection was integrated and was divided by the moles of antigen injected. The analysis using Origin software 7.0 provided three parameters: binding stoichiometry (*n*), equilibrium association constant (*K*_a_), and binding enthalpy change (∆*H*). The binding Gibbs free energy (∆*G*) was determined from the equation ∆*G* = −R*T* ln *K*_a_, and the binding entropy change (∆*S*) was determined from the equation ∆*G* = ∆*H* − *T*∆*S*.

### 4.4. DSC Experiments

DSC measurements were carried out using a VP-Capillary DSC (Malvern Panalytical, Tokyo, Japan), as described previously [27]. The scFv solution (1.0 mg mL^−1^) in the absence or presence of antigens NP-Gly and NNP-Cap, with a heating rate of 1.0 °C min^−1^. Analysis using Origin software 7.0 supplied by the manufacturer provided the thermal denaturation temperature (*T*_d_) and the calorimetric enthalpy change (∆*H*_cal_).

## Figures and Tables

**Table 1:** Folding thermodynamics of scFvs.

	CD	DSC	
	*T*_m_ (°C)	*T*_d_ (°C)	∆*H*_cal_ (kJ mol^−1^)
C6	54.2 ± 1.4	50.6 ± 0.4	494 ± 30.0
C6_R58K^H^	54.9 ± 1.9	49.8 ± 0.2	454 ± 39.5
C6_Q100E^H^	53.4 ± 2.3	46.3 ± 0.1	365 ± 22.9
C6_100aI^H^	52.4 ± 1.0	48.5 ± 0.2	416 ± 22.7
C6_Q100E^H^/100aI^H^	54.6 ± 1.0	49.8 ± 0.3	470 ± 28.9
E11	56.1 ± 1.2	51.7 ± 0.5	440 ± 5.9
E11_Δ100aI^H^	52.0 ± 2.2	45.8 ± 0.1	394 ± 8.5

The averaged values of three independent measurements with standard deviations.

**Table 2:** Thermodynamic parameters of interactions of anti-NP scFvs to NP and NNP.

	*n*	*K*_a_	∆*G*	∆*H*	*T*∆*S*
		(M^−1^)	(kJ mol^−1^)	(kJ mol^−1^)	(kJ mol^−1^)
***NP***					
C6	1.05	2.12 (±0.44) × 10^7^	−41.8	−46.1 ± 3.6	−4.3
C6_R58K^H^	0.98	8.77 (±2.87) × 10^6^	−39.5	−48.9 ± 1.1	−9.4
C6_Q100E^H^	1.02	1.43 (±0.40) × 10^7^	−40.8	−43.6 ± 0.9	−2.8
C6_100aI^H^	0.96	3.90 (±0.97) × 10^5^	−31.9	−12.3 ± 2.0	19.5
C6_Q100E^H^/100aI^H^	0.72	7.52 (±2.83) × 10^4^	−27.7	−30.7 ± 3.2	−3.0
E11 ^a^	1.02	2.17 (±0.91) × 10^8^	−47.4	−69.8 ± 2.0	−22.4
E11_Δ100aI^H^	1.02	7.30 (±0.83) × 10^5^	−33.5	−55.5 ± 1.6	−22.0
***NNP***					
C6	0.99	2.25 (±0.75) × 10^8^	−47.6	−55.0 ± 1.9	−7.5
C6_R58K^H^	0.92	3.06 (±1.04) × 10^8^	−48.3	−59.9 ± 1.2	−11.6
C6_Q100E^H^	0.94	1.80 (±1.27) × 10^8^	−46.7	−51.3 ± 2.5	−4.6
C6_100aI^H^	1.04	2.62 (±0.69) × 10^6^	−36.6	−29.3 ± 0.3	7.3
C6_Q100E^H^/100aI^H^	0.99	1.95 (±0.39) × 10^6^	−35.9	−27.5 ± 0.3	8.4
E11	1.06	4.13 (±1.90) × 10^8^	−49.0	−64.4 ± 2.8	−15.3
E11_Δ100aI^H^	1.00	2.33 (±0.70) × 10^7^	−42.0	−62.1 ± 1.9	−20.1

The averaged values of three independent measurements with standard deviations. ^a^ Data were taken from Yoshida et al. (2024) [28].

**Table 3:** Folding thermodynamics of scFvs in the presence of antigens.

	CD	DSC	
	*T*_m_ (°C)	*T*_d_ (°C)	∆*H*_cal_ (kJ mol^−1^)
***NP***			
C6 + NP	62.5 ± 0.2	67.2 ± 0.3	731 ± 29.9
C6_R58K^H^ + NP	58.1 ± 2.4	62.8 ± 0.2	654 ± 38.7
C6_Q100E^H^ + NP	56.6 ± 0.2	61.8 ± 0.3	585 ± 40.8
C6_100aI^H^ + NP	54.9 ± 1.3	53.0 ± 0.1	520 ± 21.4
C6_Q100E^H^/100aI^H^ + NP	56.4 ± 1.4	52.8 ± 0.8	584 ± 29.3
E11 + NP	66.7 ± 2.1	71.9 ± 0.1	725 ± 23.0
E11_Δ100aI^H^ + NP	54.3 ± 1.9	54.0 ± 0.2	514 ± 3.51
***NNP***			
C6 + NNP	n.d.	70.6 ± 0.1	771 ± 36.9
C6_R58K^H^ + NNP	n.d.	68.4 ± 0.0	692 ± 15.3
C6_Q100E^H^ + NNP	n.d.	66.5 ± 0.1	657 ± 59.7
C6_100aI^H^ + NNP	56.3 ± 0.3	59.3 ± 0.2	547 ± 29.4
C6_Q100E^H^/100aI^H^ + NNP	59.7 ± 1.7	59.8 ± 0.2	635 ± 21.5
E11 + NNP	70.0 ± 0.3	77.2 ± 0.0	803 ± 5.2
E11_Δ100aI^H^ + NNP	60.5 ± 1.0	59.8 ± 0.1	578 ± 10.8

The averaged values of three independent measurements with standard deviations.

## Data Availability

Data are available on request.

## Supplementary Materials

The following supporting information can be downloaded at: www.mdpi.com/article/10.3390/molecules30030532/s1, Figure S1: Far-UV CD spectra of scFvs of C6 and E11 mutants, C6_R58K^H^ (A), C6_Q100E^H^ (B), C6_100aI^H^ (C), C6_Q100E^H^/100aI^H^ (D), and E11_Δ100aI^H^ (E) in the absence (black line) or presence (broken line) of NNP-Cap in the molar ratio of 10:1 to scFv.; Figure S2: ITC profiles and diagram representing interactions between NNP-Cap and scFvs; C6_R58K^H^ (A), C6_Q100E^H^ (B), C6_100aI^H^ (C), C6_Q100E^H^/100aI^H^ (D) and E11_Δ100aI^H^ (E). Antigen solution was titrated into the scFv solution (upper). The data points were obtained by integration of the peaks in titration profiles (lower), corrected for the dilution heat, and plotted against the molar ratio. The data were fitted using nonlinear least-squares method.; Figure S3: Heat capacity curves of scFvs of C6 and E11 mutants (black line), C6_R58K^H^ (A), C6_Q100E^H^ (B), C6_100aI^H^ (C), C6_Q100E^H^/100aI^H^ (D), and E11_Δ100aI^H^ (E) in the presence of NNP-Cap in the molar ratio of 5:1 to scFv. Heat capacity curves of buffer only used for background subtraction are also indicated (gray line).
