# Supplementary material for: Affinity Maturation for Antibody Engineering: The Critical Role of Residues on CDR Loops of Antibodies in Antigen Binding
Source: Molecules. 2025 Jan 24;30(3):532. doi: 10.3390/molecules30030532 (PMC11819675; doi:10.3390/molecules30030532)
Supplement: Supplementary file 1 [file molecules-30-00532-s001.zip › molecules-3340634-supplementary.pdf]

Fig. S1

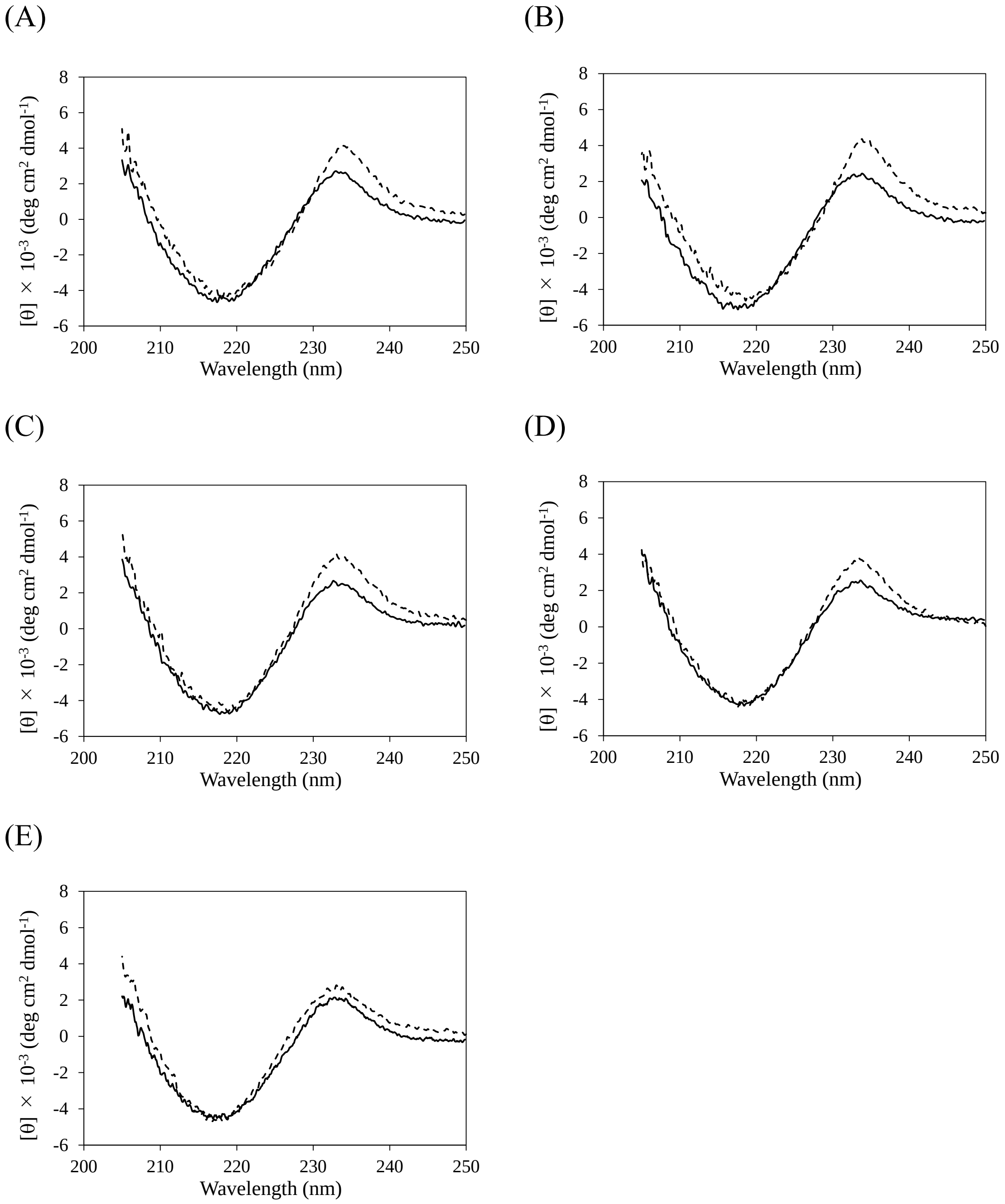

Fig. S1. Far-UV CD spectra of scFvs of C6 and E11 mutants, C6_R58K^{H} (A), C6_Q100E^{H} (B), C6_100aI^{H} (C), C6_Q100E^{H}/100aI^{H} (D), and E11_Δ100aI^{H} (E) in the absence (black line) or presence (broken line) of NNP-Cap in the molar ratio of 10:1 to scFv.

Fig. S2

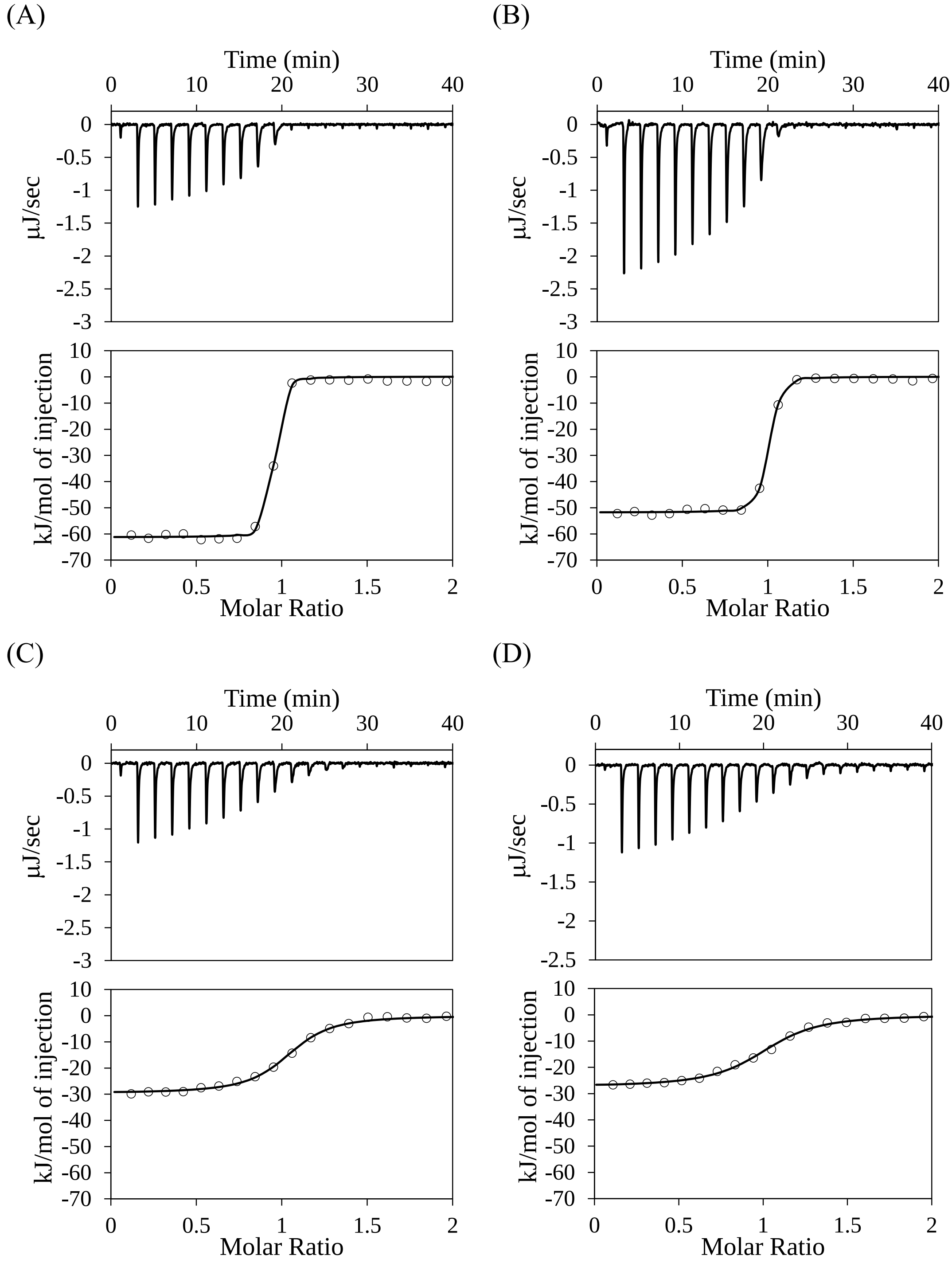

Fig. S2 (continued)

(E)

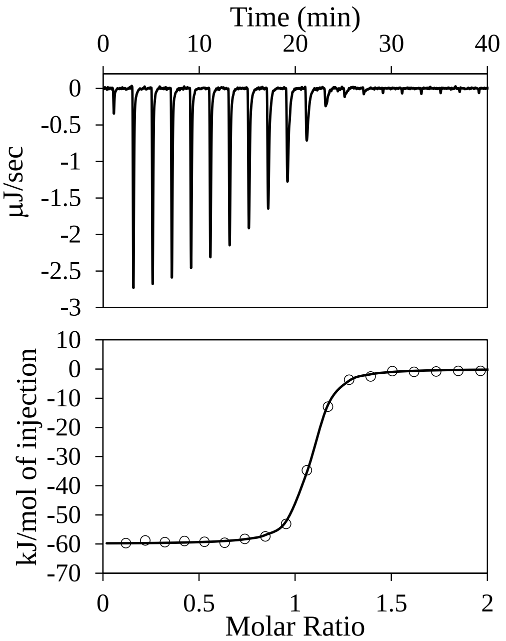

Fig. S2. ITC profiles and diagram representing interactions between NNP-Cap and scFvs; C6_R58K^H (A), C6_Q100E^H (B), C6_100aI^H (C), C6_Q100E^H/100aI^H (D) and E11_Δ100aI^H (E). Antigen solution was titrated into the scFv solution (upper). The data points were obtained by integration of the peaks in titration profiles (lower), corrected for the dilution heat, and plotted against the molar ratio. The data were fitted using nonlinear least-squares method.

Fig. S3

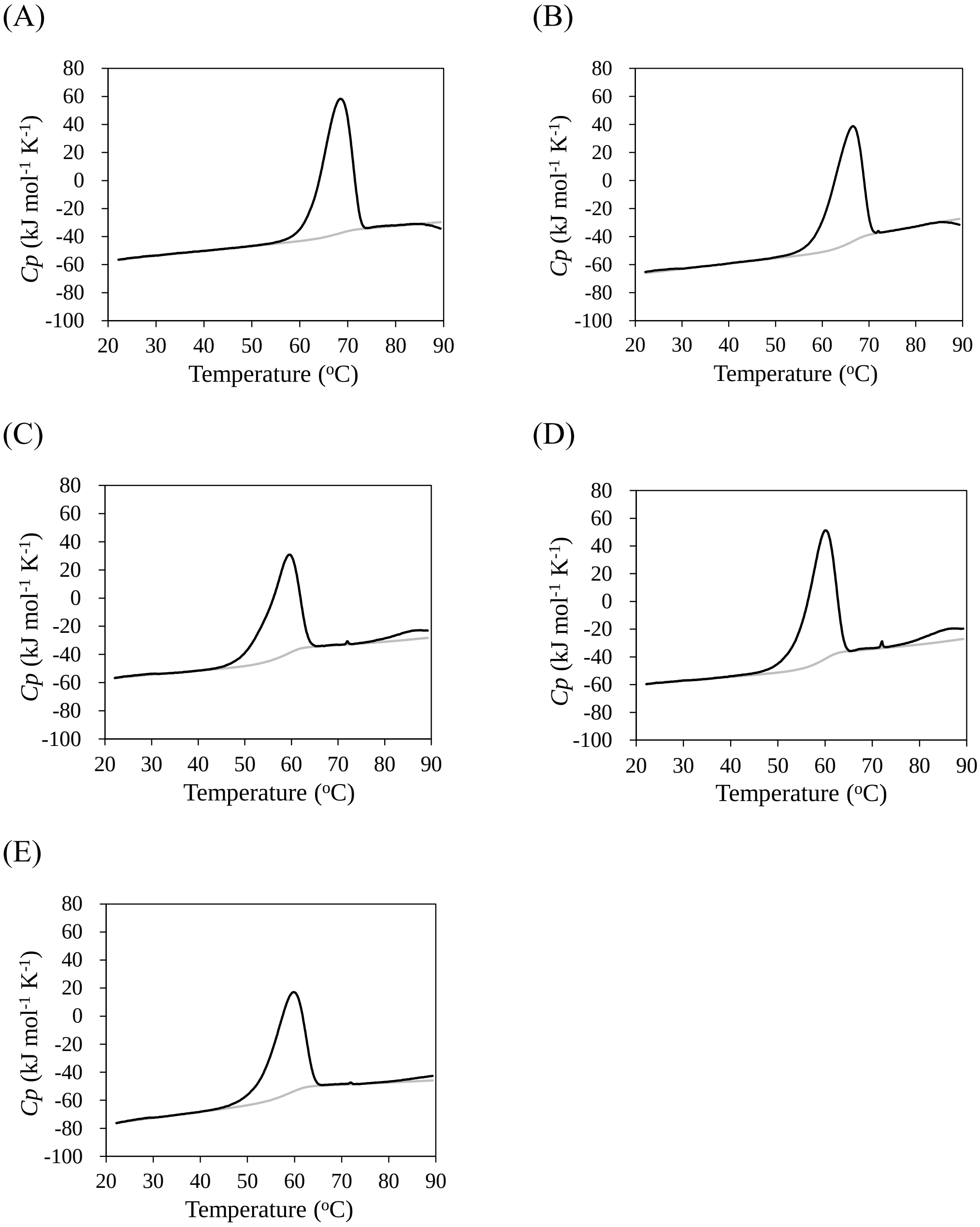

Fig. S3. Heat capacity curves of scFvs of C6 and E11 mutants (black line), C6_R58K^{H} (A), C6_Q100E^{H} (B), C6_100aI^{H} (C), C6_Q100E^{H}/100aI^{H} (D), and E11_Δ100aI^{H} (E) in the presence of NNP-Cap in the molar ratio of 5:1 to scFv. Heat capacity curves of buffer only used for background subtraction are also indicated (gray line).
